# Tuberculosis in the Central Organs of the Nervous System in Cows

**Published:** 1882-04

**Authors:** 


### EDITORIAL DEPARTMENT.

### TUBERCULOSIS IN THE CENTRAL ORGANS OF THE NERVOUS SYSTEM IN COWS.

DR. FR. ENGEL, a veterinary surgeon of Weingarten, has reported several cases of tuberculosis of the brain and cord in cows.

They are of especial interest, because they illustrate very well some of the symptoms of disease of these centres; and, furthermore, they are important because they help to prove the identity of human and bovine tuberculosis.

The first case was that of a cow eight months pregnant with her first calf. The first thing noted was that the head was held up sharply. A few weeks later, the hind legs became stiff; she then was unable to stand. The hind legs becoming completely paralyzed. The lungs seemed normal; so were also the digestive organs. Pulse, 68; temperature, 39.2° C.; respiration, 28. The right eye was somewhat wider open.

The diagnosis of tuberculosis was made, and the animal killed. The entire course of the acute symptoms had been about a fortnight. The post-mortem examination showed a tubercular inflammation of the meningitis at the base of the brain and in the anterior third of the longitudinal fissure. The tubercles were mostly gray, transparent, and the size of a pin's head. Some were, however, yellow, and of the size of a pea. In this latter respect only does the description of the appearance differ from that of the ordinary vasilor meningitis in man. The lumbar part of the spinal cord was the only portion examined. It showed also considerable tubercular meningitis.

We will not give the history of the remaining three cases (reported in the *Wochenschrift für Thierheilkunde und Viehzucht*).

It may be a help to our readers, however, to know the principal symptoms developed.

In all cases the head is held high up, and in some cases twisted to one side. The eyes have a changed appearance. They are either staring or else are directed forward, or there is some form of strabismus. Often the pupils are unequal. The limbs become stiff. The hind legs may be paralyzed, or the animal falls down, and remains on its side, with stiffened extremities.

Generally the lungs are affected at the same time, and this fact will greatly help the diagnosis.

There may, perhaps, be a few tubercles in the meninges of the brain or cord for months; but the disease is essentially an acute one, just as it is in human beings.

That the tubercles are the true tubercles of bovine tuberculosis is shown by their developing, in some cases, directly on the dura mater of the brain in the form of little pedunculated tumors; so that the German name of "pearl-disease" applies to it still when situated in this region. They also show a tendency to calcify, peculiar to the pearl-disease.

We hope to have some descriptions of these cases from our readers in this country. Bovine tuberculosis is by no means rare; but doubtless the cases in which the central nervous organs are affected are generally overlooked.

